# Evaluating multiple emission pathways for fixed cumulative carbon dioxide emissions from global-scale socioeconomic perspectives

**DOI:** 10.1007/s11027-016-9726-8

**Published:** 2016-11-10

**Authors:** Ken’ichi Matsumoto, Kaoru Tachiiri, Michio Kawamiya

**Affiliations:** 1 Graduate School of Fisheries and Environmental Sciences, Nagasaki University, Nagasaki, Japan; 2 Department of Integrated Climate Change Projection Research, Japan Agency for Marine-Earth Science and Technology, Yokohama, Japan

**Keywords:** Cumulative CO_2_ emissions, Multiple emission pathways, Climate mitigation, Socioeconomic impact, Computable general equilibrium model

## Abstract

Recent climate modeling studies have concluded that cumulative carbon emissions determine temperature increase, regardless of emission pathways. Accordingly, the optimal emission pathway can be determined from a socioeconomic standpoint. To access the path dependence of socioeconomic impacts for cumulative carbon emissions, we used a computable general equilibrium model to analyze impacts on major socioeconomic indicators on a global scale for 30–50 pathways with different emission reduction starting years, different subsequent emission pathways, and three different cumulative 2100 emission scenarios (emissions that meet the 2 °C target, the 2 °C target emissions plus 10 %, and emissions producing radiative forcing of 4.5 W/m^2^). The results show that even with identical cumulative emission figures, the resulting socioeconomic impacts vary by the pathway realized. For the United Nations 2 °C target, for example, (a) the 95 % confidence interval of cumulative global gross domestic product (GDP) is 1355–1363 trillion US dollars (2010–2100, discount rate = 5 %), (b) the cumulative GDP of pathways with later emission reduction starting years grows weaker (5 % significance level), and (c) emissions in 2100 have a moderate negative correlation with cumulative GDP. These results suggest that GDP loss is minimized with pathways with earlier emission reduction followed by more moderate reduction rates to achieve lower emission levels. Consequently, we suggest an early emission peak to meet the stringent target. In our model setting, it is desirable for emissions to peak by 2020 to reduce mitigation cost and by 2030 at the latest to meet the 2 °C target.

## Introduction

Reducing carbon dioxide (CO_2_) and other greenhouse gas (GHG) emissions is essential for preventing dangerous levels of climate change. Because it is in the interest of societies and policymakers to understand the socioeconomic impact of reducing GHG emissions, a number of analyses have been implemented that use different energy–economic and integrated assessment models (IAMs) (Clarke et al. 2009; Edenhofer et al. 2010; Jakob et al. 2012; Luderer et al. 2011; Masui et al. 2011; Matsumoto et al. 2015, 2016; Riahi et al. 2015, and many other studies). Many of these studies have used CO_2_ or GHG concentration levels or their radiative forcing effect in 2100 as climate change targets (e.g., atmospheric CO_2_ concentration at 450 ppm to meet the United Nations Framework Convention on Climate Change goal of limiting global warming to an increase of 2 °C since the pre-industrial Era).

Recently, it has been shown that cumulative CO_2_ emissions are a good indicator of climate stabilization (Allen et al. 2009; IPCC 2013; Matthews et al. 2009; Meinshausen et al. 2009; Zickfeld et al. 2009). For example, the transient climate response to cumulative carbon emissions (TCRE; IPCC 2013) is defined as the global mean surface temperature change per 1000 gigatonnes of carbon (GtC) emitted to the atmosphere. Based on a range of 0.8–2.5 °C per 1000 GtC, the TCRE can be estimated for cumulative emissions of up to 2000 GtC or the period when temperatures peak (IPCC 2013). In Integrated Assessment Modeling (IAM) studies, this indicator is also used to analyze the socioeconomic impacts of achieving a climate target (e.g., the aforementioned 2 °C target). For example, Rogelj et al. (2013a) implemented a systematic analysis of how different levels of short-term emissions (i.e., emission targets for 2020) would impact the technological and economic feasibility of achieving the 2 °C target by 2100. They used a cumulative emission budget, i.e., a value of 1500 Gt of CO_2_ (or 409 GtC) over the twenty-first century, as an indicator for staying below 2 °C, while developing different scenarios. They found that the probability of achieving the target depends strongly on the prospects of key energy technologies, as well as on the effectiveness of efficiency measures to limit the growth of energy demand. In addition, targeting lower short-term emission levels would allow the 2 °C target to be achieved under a wide range of assumptions. Riahi et al. (2015), who compared nine IAMs in their study, used a cumulative emission budget as an indicator to track this 2 °C target. They also focused on the implications of short-term policies (i.e., the national pledges issued at the United Nations Framework Convention on Climate Change Copenhagen Accord and the Cancun Agreement) on the costs and feasibility of long-term climate objectives and found that these higher near-term emissions (compared with the optimal pathways) caused significant increases in mitigation costs, increased the risk of low stabilization targets becoming unattainable, and reduced the chances of staying below the proposed temperature change target of 2 °C in case of overshoot. They also found that such pathways to 2030 would narrow policy choices. Similarly, Bertram et al. (2015) also used nine IAMs to examine how weak near-term (up to 2030) climate policies would affect the achievement of the target. They found that both the likelihood of overshooting the carbon budget and the urgency of reducing GHG emissions after 2030 increased, particularly with regard to negative emissions in the latter half of the century. They also found that much of the near-term emissions growth was a result of additional coal-fired power generation, suggesting that early retirement of coal energy and rapid increases in low-carbon technology are required. Wang et al. (2015) proposed a new scheme for carbon permit allocation considering international cooperation in climate mitigation from the perspective of equity that considers equality, historical responsibility, capability, and future development opportunities with different weights on each, based on the IAM analysis. They determined that developed countries should reduce emissions immediately, while developing countries should be allowed initially to increase their emissions. They also suggest that dynamic choice in the weights on the four equity indicators for international agreements and emissions trading for cost-efficiency are both of great importance.

Many of the above studies focus on socioeconomic impacts from a technological perspective and with regard to the viability of achieving global warming targets, while Wang et al. (2015) focus on an emissions trading scheme and its permit allocation from the viewpoint of equity. However, they do not explore the impact of taking different emission pathways on cumulative emission budgets or fixed cumulative CO_2_ emissions (FCEs). Understanding such impacts is important for society because our capacity to reduce CO_2_ emissions may vary each year, reflecting changes in factors such as technology and economic conditions. This is also important background information for policymakers required to define worldwide practices.

There are also many studies that analyze the 2 °C target from socioeconomic perspectives by considering a delay in action on emission reduction. Den Elzen et al. (2010) analyzed the costs of a delay in mitigation action and found that, although costs were lower in the short-term, they were higher in the longer term. They also noted that, if emission reductions were postponed to 2030, higher emissions in the earlier periods were not likely to be fully compensated for in later decades. Full compensation would require emission reduction rates in the coming decades that were much higher than those found in the scenario literature. Luderer et al. (2011) compared the results of three IAMs for success in achieving the 2 °C target (atmospheric CO_2_ content of 450 ppm) and showed that a delay in climate policy or restrictions to the development of low-carbon technologies could result in substantial increases to mitigation costs. They also indicated that the target would be unachievable if the delay was extended to 2030. Van Vliet et al. (2012) compared several pathways for achieving the temperature target and found that the emission pathway under the Copenhagen Accord (conditional pledges) was more costly than that of immediate full participation to achieve the target. In addition, the Copenhagen Accord (unilateral pledges), which delays emission reduction compared with the full participation scenario, reduced the probability of achieving this target. Jakob et al. (2012), who used three IAMs, analyzed the situation in which the implementation of a global climate agreement was delayed or in which major emitters participated in the agreement at a later stage. They found that the delay of a global agreement until 2020 increases the cost of mitigation to achieve 450 ppm CO_2_ by about 50 % compared with the least-cost scenario, and the delay to 2030 made the target infeasible. Kriegler et al. (2013a), which evaluated the cost and probability of achieving the 2 °C target assuming different emission levels and different long-term concentration levels in their Low climate IMpact scenarios and the Implications of required Tight emission control Strategies (LIMITS) Project, also showed similar implications. Luderer et al. (2013) analyzed the influence of a further delay in action and technology availability on implementation of the 2 °C target. They found that if emission reduction started in the early years and if low-carbon technology was fully available, the likely probability was that warming in the twenty-first century would remain below 2 °C and at moderate economic cost. However, a delay in mitigation action and the unavailability of carbon capture and storage (CCS) increased the available temperature targets by about 0.3–0.4 °C. Admiraal et al. (2015) analyzed how the timing of emission reduction affects economic costs and benefits. This study considers the aspects of not only mitigation but also of adaptation and climate damage. They found that the total costs and net benefits are greater in the gradual mitigation pathway compared with the early or delayed mitigation scenarios. Warren et al. (2013) evaluated the impact (physical and economic) of delay in mitigation action using their physical- and economic-based models and found that early, stringent mitigation would avoid a large proportion of the future impacts but not all the impacts were avoided.

These studies suggest that if mitigation action is globally delayed, the costs to achieve the target will be much higher and that the target may even become infeasible. However, other characteristics of emission pathways that achieve a certain temperature target or a cumulative emission, including emission levels at the end of the century, have not yet been analyzed.

In addition, there have been a huge number of studies on climate change mitigation policy and measures on a global scale, particularly focusing on the 2 °C target. Edenhofer et al. (2010) compared the results of five IAMs for success in achieving the 2 °C target, with different probabilities for achieving the target (at 400, 450, and 550 ppm). They found that such a temperature target was technically feasible and economically viable. They also analyzed the effect of low-carbon technologies, such as CCS, biomass, and nuclear power, on achieving the target. Den Elzen et al. (2013) analyzed abatement costs to countries for achieving an ambitious global emission reduction target by 2050 (to 50 % of 1990 emissions) considering different efforts of developed countries. They found that abatement costs would be higher for developing countries when the emission reduction targets of developed countries are smaller (less than an 85 % reduction), whereas the costs would be higher for developed countries when their target is larger (greater than an 85 % reduction). Hof et al. (2013) evaluated the emission gap for achieving the 2 °C target and the probability thereof after updating the emission reduction pledges for 2020 and the business-as-usual scenario in the Cancun Agreement. They showed that although achieving the target is possible with the pledges, high reduction rates would be required after 2020. Kriegler et al. (2013b) focused on the importance of mitigation technologies, such as CCS, nuclear power, and renewable energy in their model comparison study, and found that technologies that realized negative emissions were the most important elements for climate mitigation. Rogelj et al. (2013b) assessed the cost distribution of achieving the target under uncertainties in geopolitical, technological, social, and political factors and found that political factor (delay in mitigation action) had the largest impact on the cost. Alexeeva and Anger (2016) evaluated the economic implications, in terms of welfare and competitiveness, of linking emissions trading schemes including the Clean Development Mechanism with the 2 °C target in mind, using their computable general equilibrium (CGE) model. They suggest that integrating these schemes yields economic welfare improvement. However, while the terms of trade were improved in the countries of European Union, the opposite consequence was seen in the other countries.

Matsumoto et al. (2015) analyzed the socioeconomic impact of mitigating emissions based on an FCE for the twenty-first century, using a CGE model. They systematically developed five emission pathways based on an FCE—all pathways show emissions beginning to decline from the reference level in 2040, to attain zero by 2100. However, because these emission pathways were simple, the number of pathways was small, and only one cumulative emission was analyzed, no in-depth analysis of the relationship between the various aspects of the pathways for cumulative emissions and their socioeconomic impact was implemented.

In climate modeling research, as mentioned above, cumulative CO_2_ emissions are an important factor, indicating that a cumulative carbon emission determines the global temperature rise regardless of emission pathways taken. This means that the optimal emission pathway to achieve a global temperature target can be determined from socioeconomic perspectives. The purpose of this study is to understand if the relationship between cumulative emissions and socioeconomic factors holds true as well, in other words, to investigate the path dependence of socioeconomic impacts. To do this, we analyze the socioeconomic impact of various emission pathways under the constraint of FCEs on a global scale, using a CGE model. In particular, we examine their effects on carbon price (or marginal abatement cost), global total gross domestic product (GDP), and energy demands, as a basis for evaluating socioeconomic impacts. First, we examine the temporal features of the impacts to gain an overview of the results. We then further investigate the results of model calculation by using statistical methods. Although specific cumulative CO_2_ emissions are required to achieve a global warming target (TCRE), the pathways to achieve this target are variable and the socioeconomic impact can be different according to the selected emission pathway. Carbon pricing increases energy prices, particularly fossil fuels. This increase in prices affects the economic activity of both the industrial sector and consumers. These effects then influence indicators of whole economic activities, such as total energy demand and GDP. Thus, understanding such influences is of global societal interest. In particular, because policymakers are concerned about the socioeconomic impacts of these different policies and emission pathways when implementing climate change measures, this economic information is crucial for policymaking. By analyzing such effects, we provide policymakers with the information required for selecting a suitable emission pathway that meets the future climate stabilization target. If they know that a given emission pathway will have less negative economic impact while reducing fossil fuel consumption, they can adopt policies to achieve that pathway with greater public acceptance. In addition, by comparing three cumulative emission levels, we try to promote a better understanding of the characteristics of cumulative emissions from socioeconomic perspectives. In other words, this study is a good starting point to evaluate the cumulative emissions under various pathways from a socioeconomic perspective.

## Methods

To achieve the purpose of this study, a CGE model (Section 2.1) is used to analyze a reference scenario and multiple emission reduction pathways for three FCE scenarios (Section 2.3). In analyzing the FCE scenarios, the developed emission pathways are used as constraints when running the model. In addition, statistical methods (Section 2.2) are applied to further analyze the model results.

### CGE model

We used an economic model to analyze multiple emission pathways for given FCEs from various socioeconomic perspectives. This model is a multi-regional/multi-sectoral recursive dynamic CGE model on a global scale, with energy and environmental (GHG emissions) components. The model is based on works such as Masui et al. (2011), Matsumoto (2013), Matsumoto and Masui (2009, 2011), and Okagawa et al. (2012). As full model details are described in our previous studies, such as those of Matsumoto and Andriosopoulos (2016) and Matsumoto et al. (2016), only the major features of the model are provided here.

The model disaggregates the world into 24 geographical regions, each of which has 21 industrial sectors and a final demand sector (Table 1). In the electric power sector, a diversity of technologies, including thermal, hydroelectric, nuclear, and several types of renewable energy (see Table 1), is explicitly assumed. In addition, CCS technology can be selected as an advanced technology for thermal and biomass power generation. However, other breakthrough technologies, such as super grids, are not considered in the model. In addition, drastic changes in economic structure are not considered because these are difficult to predict. However, future energy efficiency improvement is included as an exogenous parameter as autonomous energy efficiency improvement (AEEI) as often used in this kind of model. Each industrial sector is represented by a nested constant elasticity of substitution (CES) production function, in which substitution is considered for production factors, energy sources, and intermediate inputs based on relative prices and elasticity parameters. The detailed structures are explained in Matsumoto and Andriosopoulos (2016) and Matsumoto et al. (2016).

**Table 1:** Definitions of regions and sectors in the CGE model^a^

Code	Region	Code	Commodities/sectors
AUS	Australia	[Energy]	
NZL	New Zealand	COA	Coal
JPN	Japan	OIL	Crude oil
CAN	Canada	GAS	Natural gas
USA	United States	P_C	Petroleum products
E15	Western EU countries	GDT	Gas manufacture and distribution
RUS	Russia	ELY	Electric power^b^
E10	Eastern EU countries	[Non-energy]	
XRE	Other Europe (e.g., Bulgaria)	AGR	Agriculture (e.g., rice)
KOR	Korea	LVK	Livestock (e.g., bovine cattle)
CHN	China and Hong Kong	FRS	Forestry
XRA	Other Asia-Pacific (e.g., Mongolia)	FSH	Fishery
IDN	Indonesia	EIS	Energy-intensive industries (e.g., chemical products)
THA	Thailand		
XSE	Other Southeast Asia (e.g., Malaysia)	OMN	Other mineral mining
IND	India	M_M	Metals and manufacturing (e.g., motor vehicles)
XSA	Other South Asia (e.g., Bangladesh)	FOD	Food processing (e.g., food products)
MEX	Mexico	OMF	Other manufacturing (e.g., textiles)
ARG	Argentina	CNS	Construction
BRA	Brazil	TRT	Transportation (e.g., air transportation)
XLM	Other Latin America (e.g., Chile)	CMN	Communication
XME	The Middle East (e.g., Saudi Arabia)	WTR	Water
ZAF	South Africa	OSG	Governmental services (e.g., education)
XAF	Other Africa (e.g., Egypt)	SER	Other services (e.g., insurance)

^a^This table is created based on Matsumoto et al. (2016)

^b^The electric power sector consists of thermal power (i.e., coal-, oil-, and gas-fired), hydropower, nuclear power, solar power, wind power, geothermal power, biomass power, waste power, and other renewable energy. In addition, thermal power and biomass power with CCS technology are available

Each industrial sector produces products/services delivered for international and/or domestic markets. In each domestic market, the supplied products/services are consumed as final consumption, investment, and/or intermediate inputs. For each period, the total investment demand is set exogenously to meet an assumed future economic growth rate.

The final demand sector in each region owns all production factors (capital, labor, land, and resources) and supplies them to the industrial sectors to earn income for final consumption and savings. The final demand for each product/service is determined to maximize the utility represented by a CES function.

From the activities of industrial sectors (i.e., production) and the final demand sector (i.e., final consumption) in each region, GHGs, including CO_2_, are emitted. The model is run to simulate global emission pathways between the base year (2001) and 2100 for the FCE scenarios, whereas such constraints are not applied to the reference scenario. In the model, global emissions trading is taken into account when reducing emissions compared with the reference level in the FCE scenarios. The total annual global emission allowances are equal to the global emission level in each year of the target emission pathway. Emission allowances are allocated to each region, in proportion to their projected population from the year 2050 onwards. Between the base year and 2050, regional emission allowances are set using linear interpolation between the observed emissions in the base year and the assigned emission allowances for 2050.

The model is calibrated to reproduce economic activity and energy levels in the base year, using various published data: the Global Trade Analysis Project (GTAP) 6 database (Dimaranan 2006) for economic activity levels, the Emission Database for Global Atmospheric Research v4.2 (European Commission Joint Research Centre 2011) for GHG emissions, and the International Energy Agency (IEA) energy balance tables (IEA 2009a, b) for energy.

By running the model, with the above data and the scenarios (Section 2.3), we get the outputs such as economy, energy, and emissions. The model was developed with the General Algebraic Modeling System (GAMS) software using the mathematical programming system for general equilibrium analysis (MPSGE) modeling framework.

### Statistical analysis

We applied statistical methods to implement detailed analysis for the results of model calculation to identify the results from statistical perspectives. More specifically, we implemented three analyses. First, cumulative impacts on economy and primary energy demand among the pathways are compared by scenario using boxplots.

Second, using an independent *t* test, one-way analysis of variance (ANOVA), and Tukey’s honestly significant difference (HSD) test, we determine whether statistically significant differences in the socioeconomic factors (i.e., carbon tax, primary energy (total and renewable energy), and GDP) exist among emission pathways with different emission reduction starting years. An independent *t* test is applied to the two lower cumulative emission scenarios (for all the factors), in which 2 years are compared, whereas a one-way ANOVA (for testing differences among all groups) and Tukey’s HSD test (for multiple comparison based on the one-way ANOVA) are applied to the highest cumulative emission scenario (for all the factors), in which 3 years are compared.

Finally, using correlation analysis and scatter plots, we identify the relationships between emission levels in 2100 and socioeconomic factors by scenario.

### Future scenarios

Using the CGE model, the reference scenario and three FCE scenarios were analyzed. Each FCE scenario consists of around 30–50 emission pathways. This means that one FCE “scenario” has multiple emission pathways all pathways in each scenario share the same cumulative emission.

#### Reference scenario

Before analyzing the FCE scenarios, a business-as-usual scenario (or a reference scenario) was developed. The reference scenario assumes that no policies and measures that aim to control GHG emissions are introduced.

**Fig. 1:**
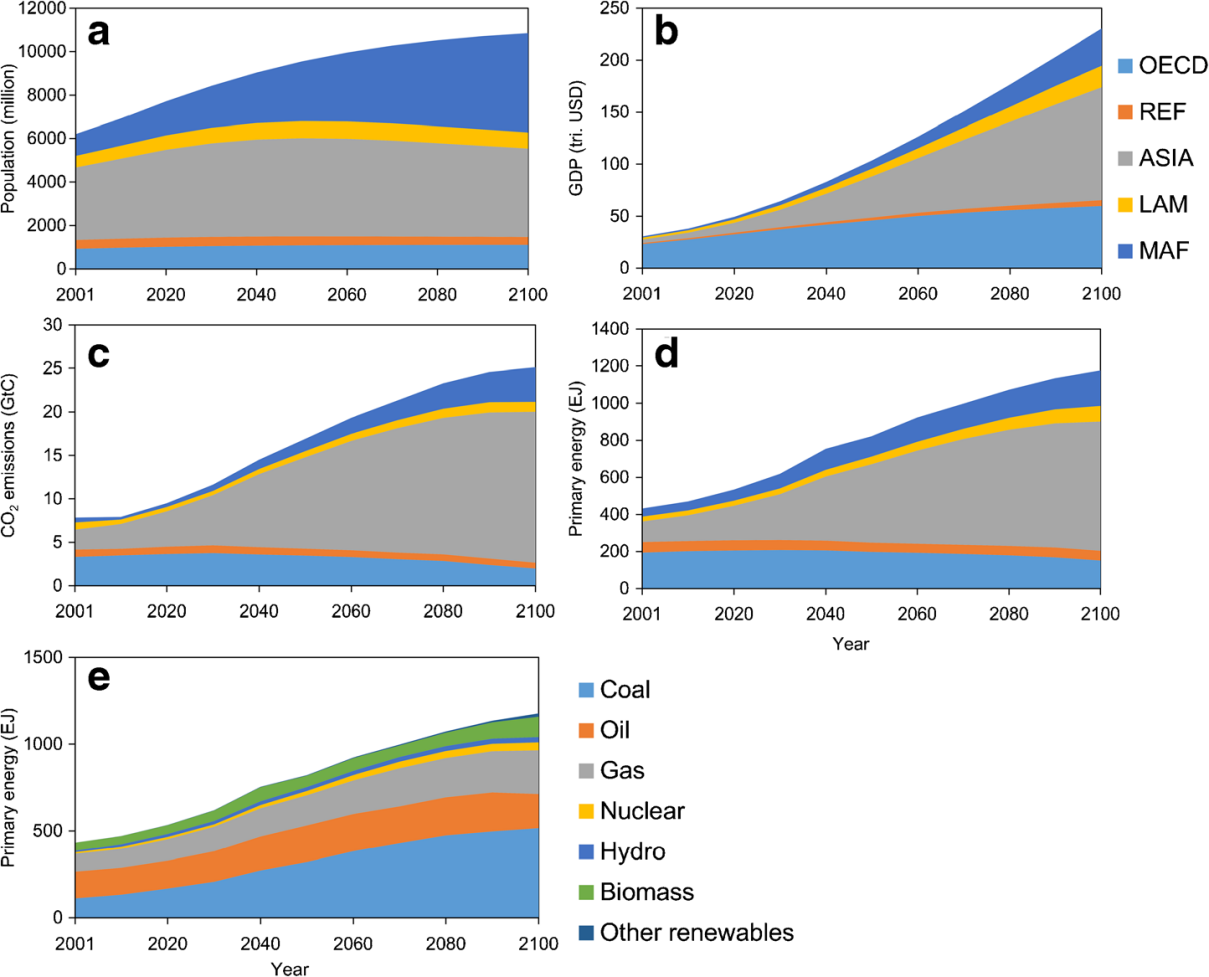
Properties of the reference scenario from the base year to 2100: **a** population, **b** GDP, **c** total CO_2_ emissions, **d** primary energy demand by region, and **e** primary energy demand by fuel type. Five regions are defined: *OECD* member states of the Organisation for Economic Cooperation and Development as of 1990, *REF* countries from the reforming economies of Eastern Europe and the former Soviet Union, *ASIA* most Asia-Pacific countries excluding the Middle East and OECD countries, *LAM* Latin American and Caribbean countries, and *MAF* Middle Eastern and African countries

Assumptions in the reference scenario are shown in Fig. 1. The details of the scenario are described in Matsumoto and Andriosopoulos (2016) and Matsumoto et al. (2016). We assumed that the global population would grow from about six billion in the base year to ten billion in 2100 (Fig. 1a). Global GDP will reach around 230 trillion US dollars (USD, Fig. 1b)^1^ and global primary energy demand will reach approximately 1180 exajoules (EJ) by 2100 (Fig. 1d, e). Globally, fossil fuel demand, particularly for coal, increases continuously during this century because of its relatively low cost (Fig. 1e). Consequently, total CO_2_ emissions increase to 25.1 GtC/year by 2100 (Fig. 1c).

#### Fixed cumulative emission scenarios

**Fig. 2:**
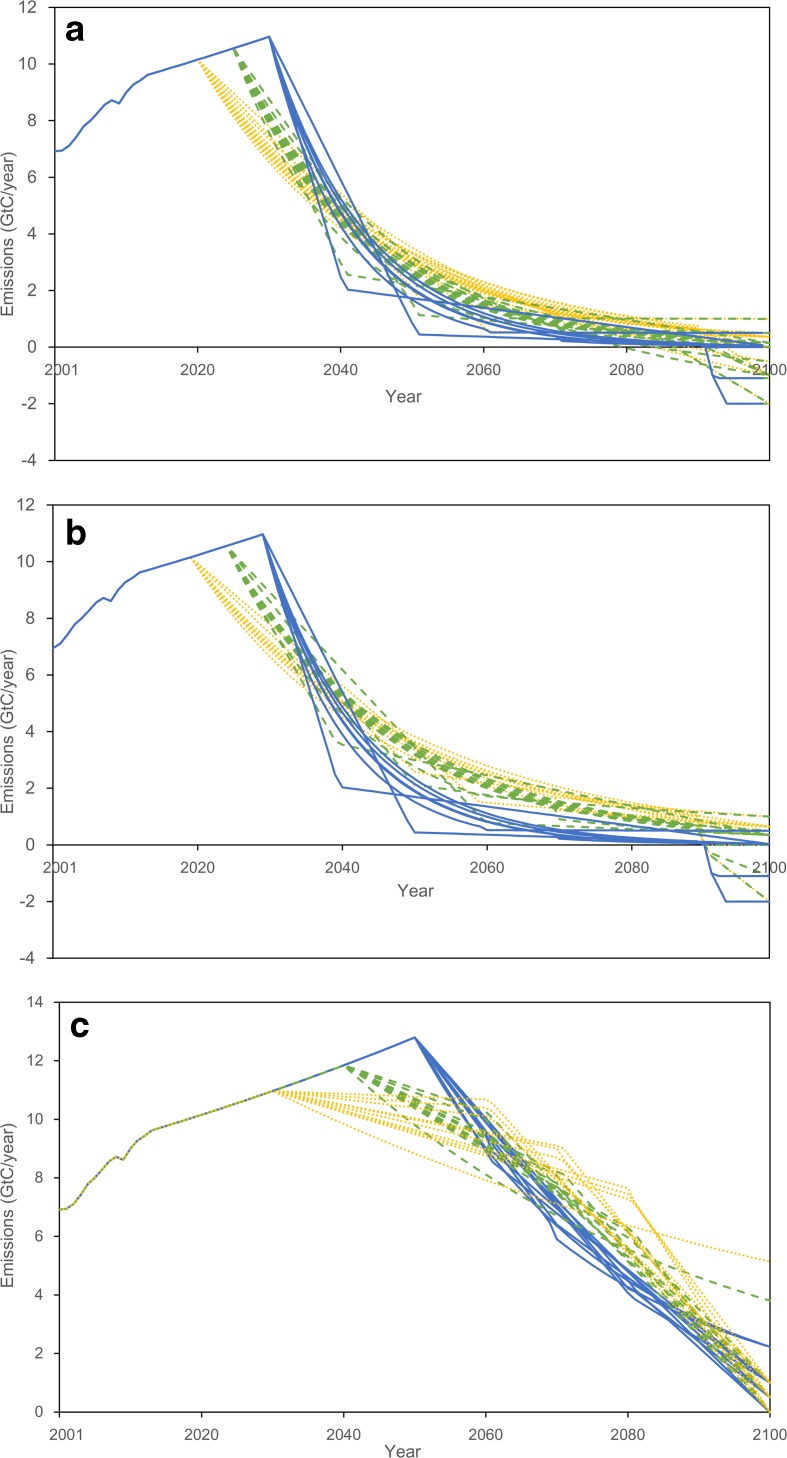
Global CO_2_ emission pathways under three fixed cumulative emission scenarios explored in this study: **a** E2d (emissions to meet the 2 °C target), **b** E2d+10p (emissions 10 % larger than E2d), and **c** E45 (emissions producing radiative forcing of 4.5 W/m^2^). In each scenario, *blue solid lines* are pathways starting emission reduction the latest (2030 or 2050), *green-dashed lines* are those starting emission reduction in the middle (2025 or 2040), and *orange-dotted lines* are those with emission reduction starting the earliest (2020 or 2040). These pathways were developed based on Table 2

The FCE scenarios are emission reduction scenarios against the reference scenario. In this study, we employ three cumulative emission scenarios in the twenty-first century to investigate the global socioeconomic impacts derived from different emission pathways used to meet the FCEs. The main target of this study is the cumulative emissions corresponding to the 2 °C target (hereafter called the E2d scenario) (Riahi et al. 2015; Rogelj et al. 2013a). The other two scenarios are cumulative emission 10 % higher than E2d and emissions corresponding to the representative concentration pathway (RCP) of emissions producing radiative forcing of 4.5 W/m^2^, which is the second lowest of the RCP scenarios (Thomson et al. 2011), for comparison and sensitivity analysis (hereafter, called the E2d+10p and E45 scenarios, respectively). The cumulative emissions of the three scenarios adopted here are 409 GtC (Rogelj et al. 2013a; Riahi et al. 2015), 450 GtC, and 819 GtC (Thomson et al. 2011), respectively, in this century. In these scenarios, various emission pathways are developed to be smooth by combining different emission reduction starting years (i.e., the peak year) and different emission levels in 2100 (Fig. 2). More precisely, emission reduction starting from 2020, 2025, 2030, 2040, and 2050 is shown. The emission levels in 2100 are those achieved by an emission reduction with constant percentages, 1, 0.5, 0, −0.5, −1, and −2 GtC. The combinations to develop emission pathways in each scenario are shown in Table 2. In the model analysis, these emissions are given exogenously as constraints rather than as being solved in the model.

**Table 2:** Scenarios and emission pathways analyzed in this study and results on cumulative impacts (GDP and primary energy demand)^a^

Scenario	Cumulative emissions in the twenty-first century (GtC)	Year emission reduction starts (year emission peaks)	Emission levels in 2100 (GtC/year)^b^	Cumulative GDP (trillion USD, 2010–2100, discount rate = 5 %)^c^	Cumulative primary energy demand (EJ)^c^
E2d	409	2020	1	1360	51,852
			0.5	1361	51,991–52,081
			Constant percentage (0.37)	1356–1361, Inf	52,009–52,107, Inf
			0	1362, Inf	52,080–52,154, Inf
			−0.5	1363	52,441
			−1	1362–1364	52,228–52,671
			−2	1364	52,611–52,643
		2025	1	Inf	Inf
			0.5	Inf	Inf
			Constant percentage (0.15)	1355, Inf	52,050, Inf
			0	Inf	Inf
			−0.5	Inf	Inf
			−1	Inf	Inf
			−2	1361	52,700–52,712
		2030	0.5	Inf	Inf
			Constant percentage (0.03)	Inf	Inf
			0	Inf	Inf
			−0.5	Inf	Inf
			−1	Inf	Inf
			−2	Inf	Inf
E2d+10p	450	2020	1	1365	52,714
			Constant percentage (0.65)	1362–1368	52,710–53,185
			0.5	1365–1367	52,737–52,810
			0	1368	52,759–52,848
			−0.5	1368	53,100
			−1	1369, Inf	52,897–53,340, Inf
			−2	1369	53,009–53,195
		2025	1	1361	52,780
			0.5	1365, Inf	52,973, Inf
			Constant percentage (0.35)	1362–1366, Inf	52,630–53,123, Inf
			0	1366	53,075–53,148
			−0.5	1365	53,527
			−1	1366–1367	53,118–53,525
			−2	1367–1368	53,235–53,472
		2030	0.5	Inf	Inf
			Constant percentage (0.03)	Inf	Inf
			0	Inf	Inf
			−0.5	Inf	Inf
			−1	Inf	Inf
			−2	Inf	Inf
E45	819	2030	Constant percentage (5.14)	1387	60,904
			1	1387	61,824–61,910
			0.5	1387	61,949–62,031
			0	1387	61,966–62,040
		2040	Constant percentage (3.80)	1387	60,891
			1	1387	61,463–61,778
			0.5	1387	61,560–61,930
			0	1384–1387	61,629–62,060
		2050	Constant percentage (2.23)	1385–1387	61,798–61,835
			1	1385–1387	61,843–62,120
			0.5	1386–1387	61,967–62,011
			0	1386–1387	62,028–62,061

^a^Emission pathways are a combination of different start years of emission reduction and emission levels in 2100 under three cumulative emissions. There are multiple pathways for the same emission reduction starting years and emission levels in 2100 (i.e., different emission pathways in between)

^b^“Constant percentage” means the emission level in 2100 is the same as if emissions are reduced at a constant percentage from the year emission reduction begins

^c^“Inf” in the cells means infeasible pathways

As explained in Section 3, some emission pathways were not feasible (i.e., the pathways were not solved by the model).

## Results and discussion

In achieving the emission pathways in the three scenarios, the model assumes that emissions are reduced cost effectively through emissions trading on a global scale, as described in Section 2.1. Among the various socioeconomic factors that can be analyzed using the CGE model (e.g., GDP, welfare, consumption, trade, investment, and energy supply and demand), this study focuses on carbon price, GDP, and energy demand on a global scale because they are suitable indicators for observing the socioeconomic impacts linked to reducing emissions and are often used in this type of research (e.g., Clarke et al. 2009; Masui et al. 2011; Matsumoto et al. 2016; Thomson et al. 2011). Section 3.1 shows the overall features of the results of the model calculations. Section 3.2 then shows the detailed analysis of path dependence of the results. Here, we focus on the global-scale results. However, some regional-scale results are provided in Appendix 1.

### Temporal features of global socioeconomic impacts

**Fig. 3:**
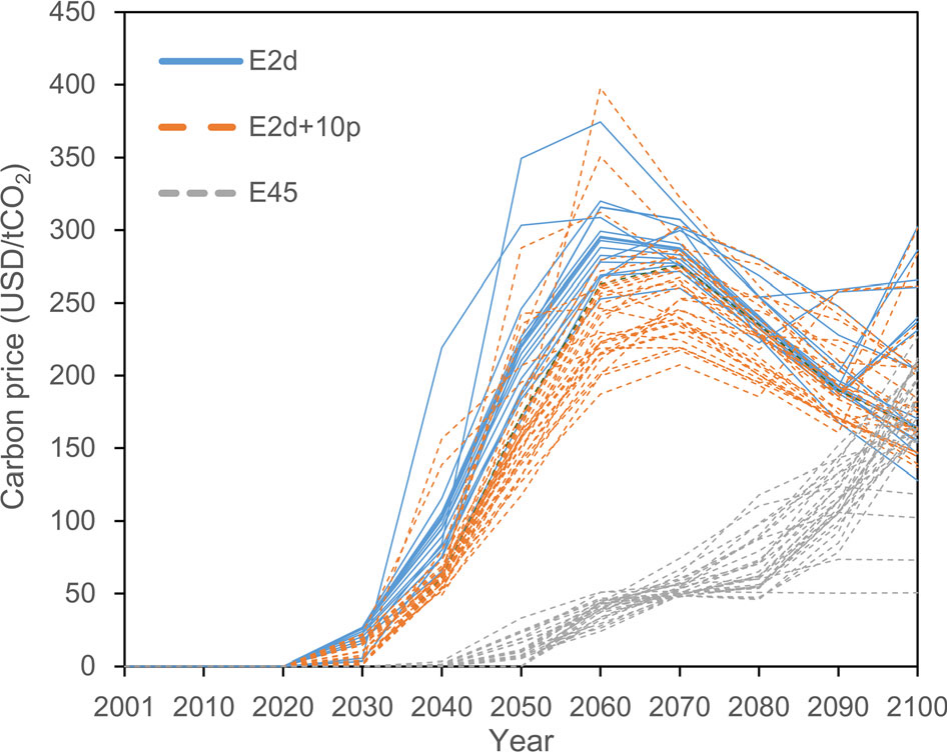
Transition of carbon prices for emission pathways of the three scenarios

Figure 3 shows carbon prices. A unified global carbon price for each year can be given because global emissions trading is modeled. The prices in the lower cumulative emission scenarios tend to be higher, which is necessary for further reduction of CO_2_ emissions. In the two lower emission scenarios (E2d and E2d+10p), prices increase sharply from the period when emission reduction starts and peak at around 2060–2070. Such changes in the carbon price are due to multiple factors, such as emission reduction, technological factors (e.g., costs and availability of low-carbon technology), and economic structure (e.g., substitutability among production factors and intermediate inputs). However, emission reduction is the most influential factor (Matsumoto et al., 2016), because a carbon price is a shadow price of CO_2_ emissions. During the periods of sharp increases in the carbon price, rapid emission reductions are observed in a global level (Fig. 2), whereas emissions continuously increase in the reference scenario (Fig. 1). To implement such emission reductions, sharp increases in the price of carbon are required. The carbon prices in the two scenarios decline after peaking in mid-century. Prices do not need to be increased further in these scenarios because the yearly amount of emission reduction in the later periods is more moderate than in the earlier periods and because energy efficiency, expressed as AEEI in the model, is improved year by year while more advanced technology (i.e., CCS technology) becomes available in the later periods.

However, in the E45 scenario, prices rise gradually over time during the twenty-first century, along with increases in emission reductions compared with the reference scenario. This is due to its slower and more linear emission pathways compared with the two lower emission scenarios.

There are also differences in carbon prices among emission pathways in each FCE scenario. However, these differences are smaller than those observed among FCE scenarios. This also indicates that emission reduction is the most influential factor in determining carbon prices, which in turn affect energy demand and GDP (economic activity) through increases in energy prices.

**Fig. 4:**
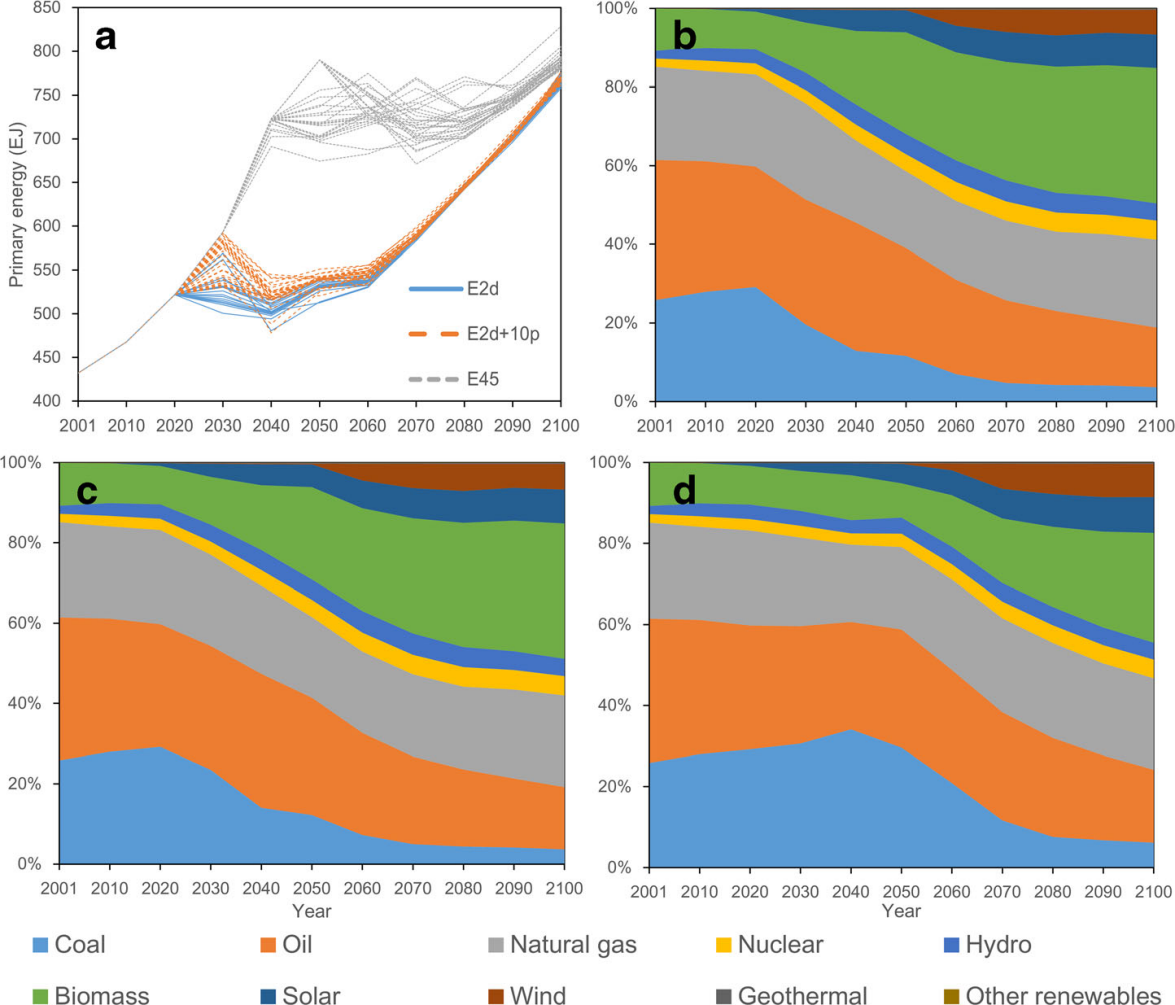
Transition of primary energy demand for emission pathways of the three scenarios and the structure of primary energy for the selected pathway from each scenario: **a** the total primary energy demand for all the pathways, **b** the structure for E2d (emission reduction starting from 2020 with a constant reduction rate), **c** the structure for E2d+10p (emission reduction starting from 2020 with a constant reduction rate), and **d** structure for E45 (emission reduction starting from 2040 with a constant reduction rate)

Global primary energy demand for the three scenarios (Fig. 4a) is lower than for the reference scenario (Fig. 1d, e) and that of the lower emission scenarios tends to be even lower. Primary energy demand increases as CO_2_ emissions increase. After emission reduction begins, primary energy demand also declines temporarily. The decrease in primary energy demand is greater for pathways that involve greater emission reductions. However, this decrease does not continue; rather, global primary energy demand increases again toward the end of the century, although emissions continuously decrease after the peaks. The differences in primary energy demands among the scenarios and among the pathways are larger immediately after emission reductions start, corresponding to the degree of emission reduction. However, they shrink over time. Once emission reduction begins, the global economy adapts to the low-carbon world (e.g., with the introduction of renewable energy). Thus, the differences in primary energy demand among the pathways and among the scenarios lessen over time. As with the carbon prices, there are differences in primary energy demand among the pathways in each FCE scenario, but the differences are smaller than those observed among the scenarios.

In all of the scenarios, the share of energy from fossil fuels, particularly coal, declines considerably during the emission reduction phase (Fig. 4b–d), in contrast with the reference scenario (Fig. 1e). Indeed, the share of energy from renewable sources, including hydropower and biomass energy, increases. Among the scenarios, on a global scale, the increase in the share of energy from renewable sources and decrease in the share of energy from coal are larger in the E2d and E2d+10p scenarios (53–57 and 3.0–3.8 %, respectively, in 2100 for the E2d scenario and 52–57 and 3.1–3.9 %, respectively, in 2100 for the E2d+10p scenario) than in the E45 scenario (46–54 and 3.6 %–7.9 %, respectively, in 2100). In all the scenarios, biomass energy accounts for the greatest share in renewable energy. Furthermore, CCS technology plays an important role for emission reduction, particularly to achieve the very low emissions in E2d and E2d+10p. In these scenarios, thermal power with CCS technology is employed, and thermal power without CCS is phased out by the end of the century. CCS technology is also used with biomass power in the model, and although biomass power without CCS continues to be used in these scenarios, biomass power with CCS occupies more than 99 %. Such transitions in the primary energy structure allow global primary energy demand to increase in the emission reduction phase.

**Fig. 5:**
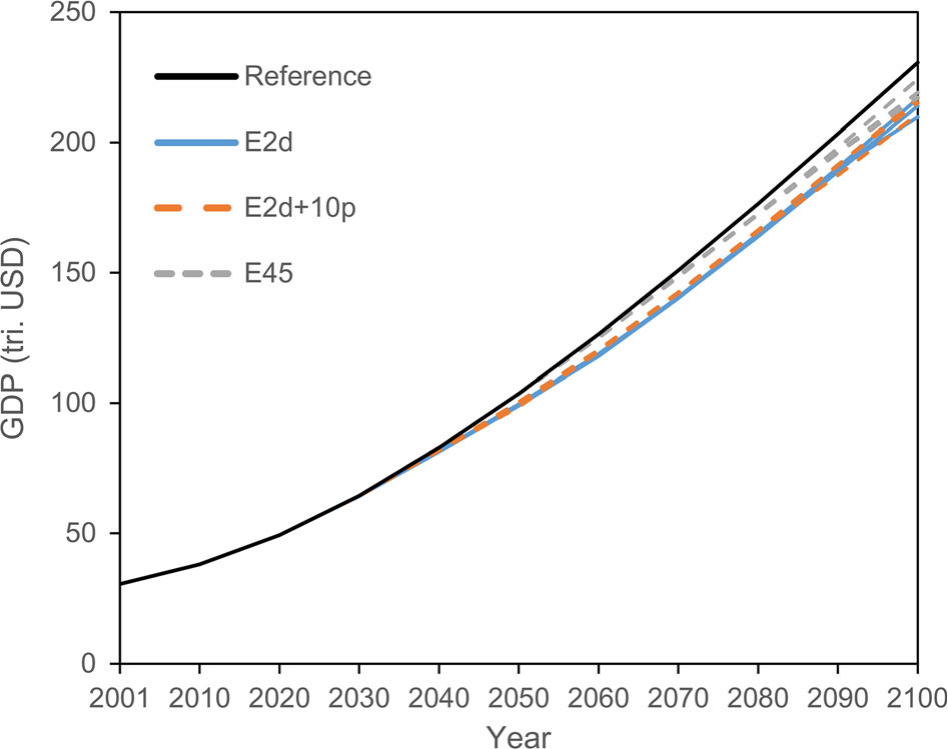
Transition of GDP for the reference scenario and three selected emission pathways from each fixed cumulative CO_2_ emissions (FCE) scenario. Pathways whose GDP levels in 2100 are in the 5th, 50th, and 95th percentile in each FCE scenario are selected

**Fig. 6:**
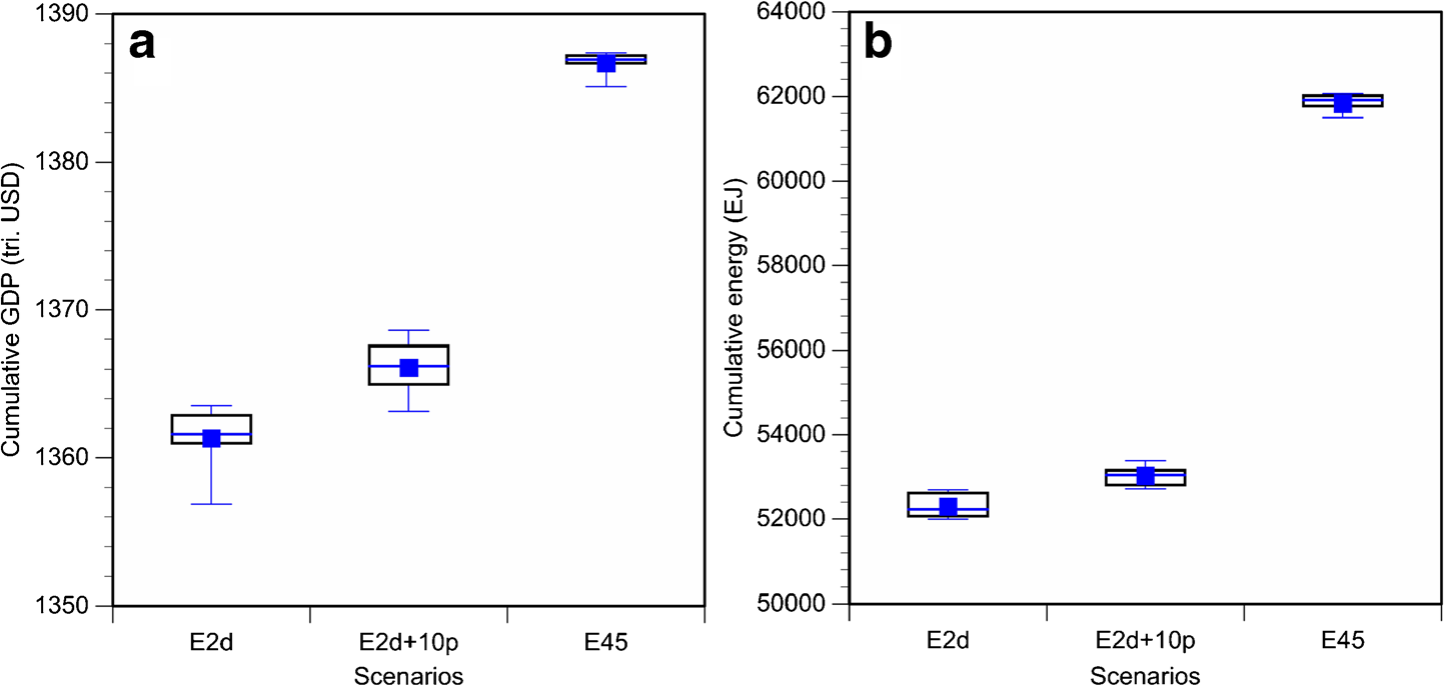
**a** Cumulative GDP (discount rate = 5 %) and **b** cumulative energy from 2010 to 2100 for the three scenarios. Three *bars* in the box show lower quartile, median, and upper quartile. *Squares* in the box show the mean. *Lower and upper whiskers* show 10th and 90th percentile, respectively.

**Fig. 7:**
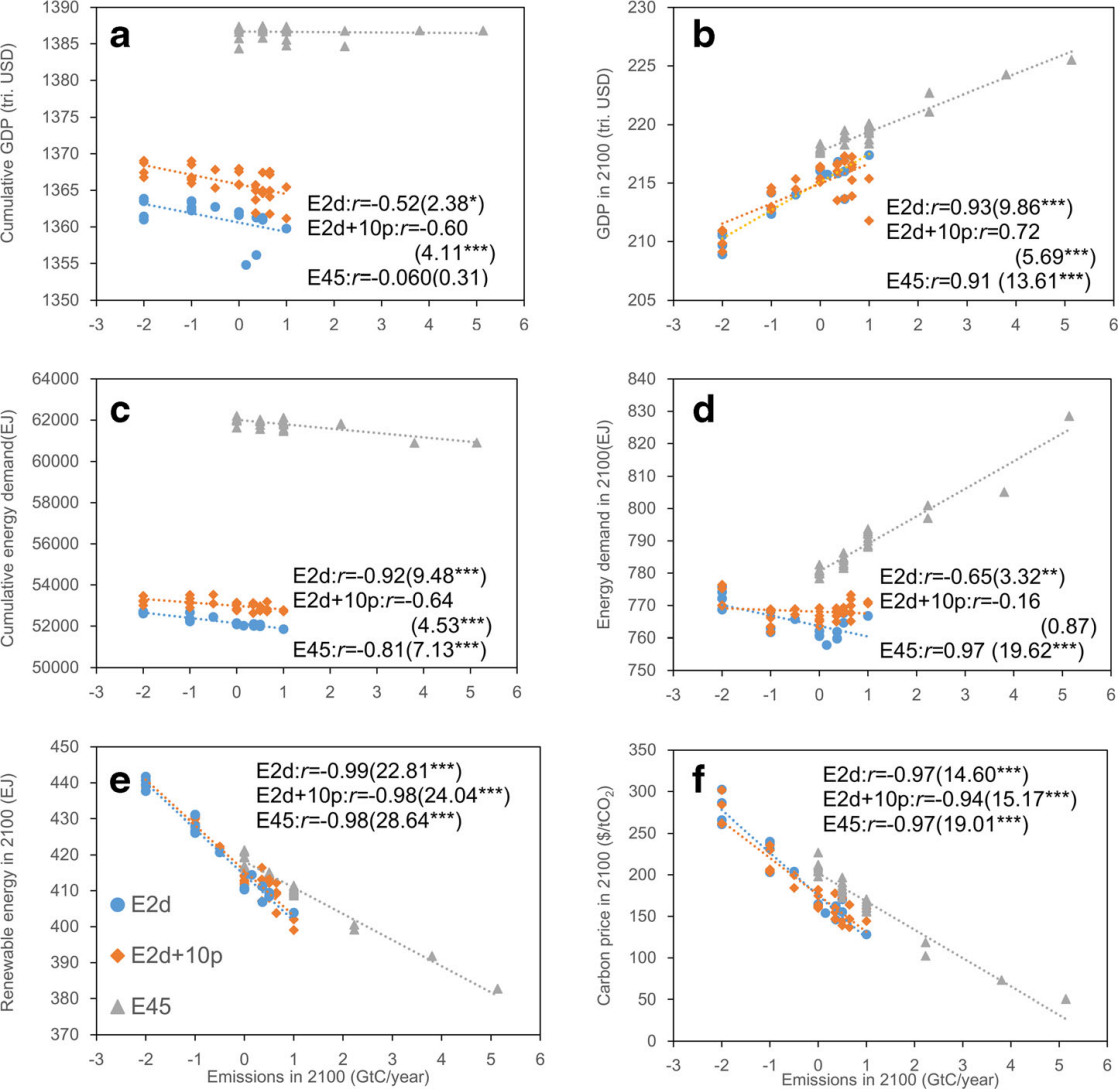
Scatter plots and correlation coefficients (*r*) related to emission levels in 2100: **a** cumulative GDP, **b** GDP in 2100, **c** cumulative energy demand, **d** energy demand in 2100, **e** renewable energy use in 2100, and **f** carbon price in 2100. The values in *parentheses* are *t* values for test for non-correlation (*one asterisk*, 5 % significance level; *two asterisks*, 1 % significance level; *three asterisks*, 0.1 % significance level). *Dotted lines* in the figures show approximate lines

Global GDP is smaller in the three FCE scenarios than in the reference scenario, although GDP continuously increases during the twenty-first century for all the scenarios (Fig. 5). The impact of emission reductions on GDP is smaller in earlier years and increases over time, reflecting emission reduction patterns. As a result, in 2100, GDP is 4.0–9.1 % smaller than in the reference level, depending on emission pathways and scenarios. Comparing GDP levels among the scenarios, those with higher cumulative emissions tend to be higher. However, as shown in Fig. 5, there is a possibility that GDP under the E2d scenario would become larger than that in the E2d+10p scenario at the end of the century (see also Fig. 6a). This is due to the emission levels in these periods—emissions in 2100 in the E2d+10p scenario are smaller than those in the E2d scenario in some pathways (see also Fig. 7b showing the correlation between emission levels in 2100 and GDP in 2100).

**Fig. 8:**
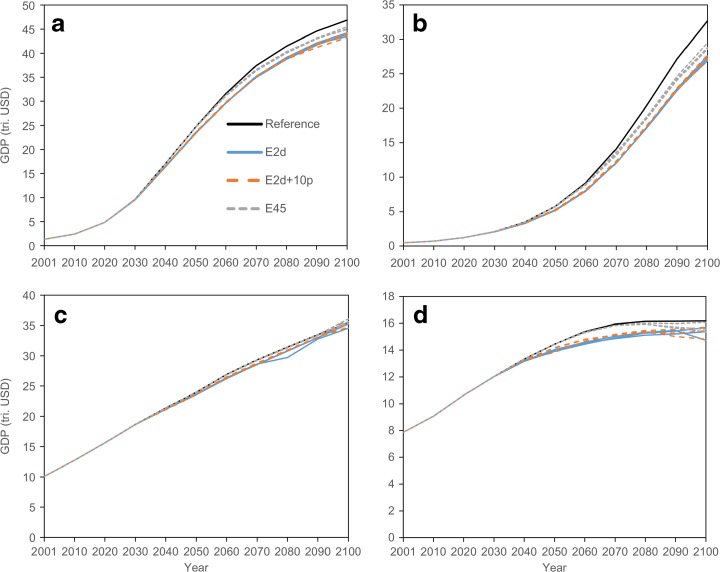
Transition of GDP for the reference scenario and three selected emission pathways from each fixed cumulative CO_2_ emissions (FCE) scenario. Pathways whose GDP levels in 2100 are in the 5th, 50th, and 95th percentile in each FCE scenario are selected. **a** China, **b** India, **c** the USA, and **d** European Union

Higher carbon prices drive up energy prices, particularly carbon-intensive energy, causing a decline in energy (fossil fuels) demand. However, substitution mechanisms would occur among production factors, energy sources, and intermediate inputs (Matsumoto et al. 2016; Fig. 8). These effects are taken into account using CES production functions within the model. This means that the production factors, energy sources, and intermediate inputs with relatively low prices are used for economic activities in the assumed elasticity parameters. Thus, the increase in carbon prices does not contribute directly to the decline in GDP; rather, the influence of this increase is reduced by these substitution mechanisms, causing differences in the GDPs among the pathways and scenarios to be smaller than differences in their carbon prices and primary energy.

### Detailed analysis of three emission reduction scenarios

The above results suggest that not only do different cumulative emissions have different effects on socioeconomic conditions (lower emissions tend to have larger global socioeconomic impacts), emission pathways with identical cumulative emissions also have different impacts on a global scale. Therefore, we examine the results more in detail to understand global socioeconomic implications under the different emission pathways realized in each cumulative emission scenario and to see if path dependence in socioeconomic impacts exists.

First, although the cumulative emissions are identical, the resulting impacts are generally different in the realized pathways (Fig. 6). The 95 % confidence interval of cumulative global GDP (Fig. 6a) for the E2d scenario is 1355–1364 trillion USD, whereas the impacts for the other two scenarios are 1362–1369 trillion USD (E2d+10p) and 1385–1387 trillion USD (E45) (c.f., 1392 trillion USD for the reference scenario). Note that cumulative global GDP is calculated based on the net present value (NPV) from 2010 to 2100, using a discount rate of 5 %, which is used in similar studies (e.g., Clarke et al. 2009; Riahi et al. 2015). Basically, the cumulative GDP of lower cumulative emissions is smaller, although there is a possibility of the inversion in cumulative GDP levels if the cumulative emissions are close (the E2d and E2d+10p scenarios in this case).

With regard to cumulative global total primary energy (Fig. 6b), the 95 % confidence intervals are 51,908–52,708 EJ (E2d), 52,693–53,525 EJ (E2d+10p), and 60,900–62,144 EJ (E45) (c.f., 77,182 EJ for the reference scenario). The relationship between cumulative emissions and cumulative energy is similar to the relationship with cumulative GDP shown above. The observed differences among the pathways within a scenario are small compared with those among cumulative emissions.

The emission reduction starting year (or the peak year of emissions) affects cumulative GDP. For example, comparing the cumulative GDP of the emission pathways with emission reduction starting years of 2020 and 2025 (the E2d scenario), the latter is significantly smaller than the former (Table 3). Similarly, in the E2d+10p and E45 scenarios, the pathways in which emission reduction begins later show significantly smaller cumulative GDP levels. Note that the multiple comparison using Tukey’s HSD test for the E45 scenario, which compares three groups, suggests that cumulative GDP levels between the emission reduction starting years of 2030 and 2050 are significantly different (5 % significance level). However, the differences in cumulative GDP between the starting years of 2030 and 2040 and those of 2040 and 2050 are not significant. These results are not affected even if smaller discount rates are applied (the *p* values are, however, different). However, significant differences were not observed for the other economic and energy indicators (cumulative energy demand and GDP, energy demand, renewable energy use, and carbon price in 2100) analyzed in the study (see Table 3), except for cumulative energy demand under the E2d+10p scenario.

**Table 3:** Independent *t* test and one-way ANOVA for different starting years of emission reduction

	Scenarios	Average^a^	Standard deviation^a^	*t* or *F* values^b^
Cumulative GDP (5 % discount rate, trillion USD)^c^	E2d	1361.8/1359.1	2.0/3.7	1.85^*^
	E2d+10p	1366.9/1365.3	2.1/1.7	2.34^*^
	E45	1387.3/1386.7/1385.9	0.19/0.83/0.84	9.18^***^
GDP in 2100 (trillion USD)	E2d	214.1/212.0	2.5/3.3	1.28
	E2d+10p	215.1/214.1	2.1/2.2	1.37
	E45	219.3/218.8/219.7	2.3/2.3/1.5	0.42
Cumulative energy demand (EJ)	E2d	52,247.0/52,477.8	260.0/396.3	0.80
	E2d+10p	52,905.2/53,131.2	198.9/239.1	2.62^**^
	E45	61,863.2/61,660.5/61,953.2	354.3/334.2/112.0	2.52
Energy demand in 2100 (EJ)	E2d	765.2/766.9	4.6/8.3	0.52
	E2d+10p	768.9/767.4	2.8/3.5	0.89
	E45	789.3/786.7/788.9	14.6/7.7/7.6	0.18
Renewable energy use in 2100 (EJ)	E2d	419.7/431.0	12.6/14.4	0.92
	E2d+10p	417.2/419.6	12.0/12.1	0.20
	E45	411.6/411.8/411.1	7.7/7.9/10.6	0.02
Carbon price in 2100 (USD/tCO_2_)	E2d	197.7/235.3	50.5/71.3	0.57
	E2d+10p	177.0/194.0	43.3/43.7	0.59
	E45	173.8/176.9/161.4	47.1/42.8/34.5	0.35

^a^The values are in ascending order of the emission reduction starting year (the first one is the earliest). In the E45 scenario, three different starting years exist

^b^In this statistical hypothesis test, the null hypothesis is that each factor (by scenario) is the same between the two or three starting years of emission reduction. A *t* test is applied to the E2d and E2d+10 % scenarios, in which two points are compared, whereas a one-way ANOVA is applied to the E45 scenario, in which three points are compared (*one asterisk*, 5 % significance level; *two asterisks*, 1 % significance level; *three asterisks*, 0.1 % significance level)

^c^They also become significant for other discount rates

There are infeasible emission pathways (i.e., pathways not solved by the model) in the lower emission scenarios (those with later reduction start years: see also “Inf” in Table 2). Emission reduction after 2030 is infeasible for all the pathways of the E2d and E2d+10p scenarios. These infeasible pathways are due to strict emission reductions as a result of delayed mitigation.

With regard to the relationship between emission levels in 2100 (the end of the model run) and socioeconomic impacts in the twenty-first century on a global scale, emission levels have a moderate negative correlation with cumulative GDP in the two lower emission scenarios (correlation coefficient *r* = −0.52 (E2d) and −0.60 (E2d+10p); Fig. 7a). In addition, a strong negative correlation is observed with cumulative energy demand, except for the E2d+10p scenario showing a moderate negative correlation (*r* = −0.64 to −0.92; Fig. 7c). Lower emissions in 2100 are brought about by higher emissions in the earlier periods. As Fig. 4a indicates, energy demand in earlier periods (soon after emission reduction begins) is largely affected by emission reductions, whereas the impact decreases over time. Thus, the cumulative energy demand under pathways with lower emissions in the later periods (i.e., higher emissions in the earlier periods) tends to be larger.

With regard to the relationship between emission levels and their impacts, both in 2100, there is a strong negative correlation between the emission levels and carbon price (*r* = −0.94 to −0.97; Fig. 7f). This is because lower emissions in any particular year result in higher marginal abatement costs in the same year.

In addition, there is a strong correlation between emission levels in 2100 and global GDP in 2100 (Fig. 7b), with the correlation coefficient *r* = 0.93 for the E2d scenario being the strongest. Increases in carbon price affect all economic activities. As a result, GDP correlates with emission levels. However, because GDP is affected not only by emission levels but also by other factors, as mentioned above, the correlation coefficients are smaller than those for carbon prices.

Similarly, emission levels in 2100 have a strong negative correlation with global renewable energy use in 2100 (*r* = −0.98 to −0.99; Fig. 7e). This is because larger emission reduction requires a greater reduction of fossil fuel use and increased renewable energy use through higher carbon prices. However, global total primary energy demand shows different features from renewable energy use (Fig. 7d). Emission levels in 2100 have a strong positive correlation (*r* = 0.97) with primary energy demand in 2100 for the E45 scenario, whereas such strong correlations are not observed in the other scenarios (*r* = −0.65 (E2d) and −0.16 (E2d+10p)). However, different patterns are observed between positive and negative emission cases. Analysis of the two scenarios by separating the data by positive/negative emissions (zero emissions are included in the negative side) suggests that a strong/moderate negative correlation is seen in the negative emission part (*r* = −0.84 for the E2d scenario and *r* = −0.63 for the E2d+10p scenario), whereas a strong/moderate positive correlation is seen in the positive emission part (*r* = 0.77 for the E2d scenario and *r* = 0.52 for the E2d+10p scenario). The latter feature is consistent with the E45 scenario, in which a strong positive correlation is seen. As in the positive emission part (in the two lower emission scenarios) and the E45 scenario, it is reasonable that lower emissions are achieved by reducing energy use. However, in the negative emission part (seen in the two lower emission scenarios), the relationship is opposite to the above. To achieve negative emissions in 2100, it is necessary to increase the use of biomass energy with CCS technology globally, as this is the only option that can offset CO_2_ emissions from other sources in the model. Such inversion occurs because biomass energy with CCS technology requires an additional energy input for capturing and storing CO_2_ (Matsumoto et al. 2016).

In the FCE scenarios, both global GDP and energy demand (total and renewable energy) increase toward the end of the century. However, even though the cumulative emissions are identical, the resulting socioeconomic impacts are generally different depending on the pathway. The difference in the emission reduction starting year affects cumulative GDP. Cumulative GDP tends to be greater if the starting year is earlier; pathways with very late starting years can be infeasible. Furthermore, emission levels in 2100 have a strong/moderate (positive or negative) correlation with the socioeconomic factors highlighted in this study. Cumulative GDP has a moderate correlation with emissions in 2100 for the two lower emission scenarios.

These results suggest that starting emission reduction earlier and achieving lower emissions at the end of the century will contribute to higher cumulative global GDP and less adverse economic impact. This also indicates that, rather than a rapid emission reduction (starting emission reduction later to achieve lower emissions in 2100) or a slow emission reduction (starting emission reduction earlier and achieving a higher emission level in 2100), moderate emission reduction taken between the emission reduction starting year and 2100 will achieve emission targets. These results indicate that path dependence is observed in socioeconomic impacts but that its effects are not large.

## Conclusion

Because TCRE was nearly constant, future temperature increase is strongly correlated with cumulative CO_2_ emission levels, regardless of the emission pathway. Hence, to determine the target emission pathway for a given climate stabilization target, for example, an acceptable temperature rise, we require an understanding of how dependent the global economy is on the emission pathway. In this study, we analyzed socioeconomic impacts of various emission pathways for three cumulative CO_2_ emissions, corresponding to the emissions required to meet the 2 °C target, emissions 10 % higher than 2 °C target emissions, and emissions producing radiative forcing of 4.5 W/m^2^ in 2100, using a CGE model. We also applied statistical methods to further analyze the features of the pathways. Global socioeconomic impacts would be different depending on the emission pathway selected even if the cumulative emissions were the same. The key global-scale findings of this study are as follows:

- Cumulative economic impacts were different depending on the pathway. For example, the 95 % confidence interval of cumulative global GDP in NPV for the E2d scenario was 1355–1364 trillion USD, whereas it was 1362–1369 trillion USD and 1385–1387 trillion USD for the E2d+10p and E45 scenarios, respectively.
- Cumulative GDP for pathways with later emission reduction start years was significantly smaller than that in pathways with earlier emission reduction start years. Thus, starting emission reduction earlier is more effective to achieve higher economic levels in this century. In addition, there were infeasible pathways in the lower emission scenarios when the starting year was very late. In particular, emission pathways that start reduction after 2030 were totally infeasible for the E2d and E2d+10p scenarios.
- Emission levels in 2100 had correlations (strong/moderate and positive/negative) with various socioeconomic factors (i.e., carbon price, primary energy demand, and GDP in this study), with a few exceptions. In the E2d scenario, emissions in 2100 were negatively correlated with cumulative GDP and cumulative energy demand. Thus, lower emission levels in 2100 would be more effective in realizing higher GDP in this century.
- From the above findings, it is expected that starting emission reduction earlier and achieving lower emissions with moderate emission reduction pathways in between will contribute to higher cumulative global GDP.

As shown in this study, emission reduction will negatively affect the global economy compared with the level of the reference scenario. However, pathways with earlier emission reduction (i.e., earlier emission peak) followed by a moderate rate of reduction achieving lower emission levels will minimize the economic impact in this century. Furthermore, in our model setting, 2020 is a desirable point for peak emissions to reduce mitigation cost, and emissions must peak by 2030 at the latest to meet the 2 °C target. Although the Paris Agreement was adopted at the 21st Conference of the Parties to the United Nations Framework Convention on Climate Change, the Intended Nationally Determined Contributions in the agreement are insufficient to achieve the early emission peak in the global level (Climate Action Tracker 2015). Thus, global talks to promote action (at the national and global level) to realize greater emission reductions and an earlier emission peak (e.g., emissions trading, carbon tax, and technological development and dissemination) are urgent for minimizing long-term economic impacts. However, our study results show that rapid emission reduction is unlikely to be necessary, while a moderate emission reduction pathway achieving lower (negative) emissions by the end of the century is better from an economic perspective. Moderate emission reduction would allow society to adapt more easily to the new low-carbon economy. For example, primary energy demand, which is a driver of economic activities, will not be greatly affected by moderate emission reduction. Renewable energy, meanwhile, is an indispensable but expensive technology for realizing a low-carbon society that can be introduced gradually. Furthermore, during the moderate reduction phase, society will have time to develop new low-carbon technology and to develop strategy for the eventual zero or negative emission world.

## Figures and Tables

**Fig. 9:**
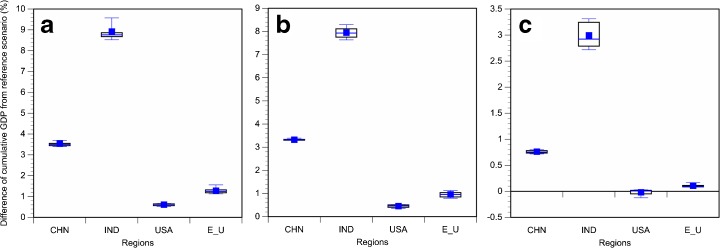
Cumulative GDP (discount rate = 5 %) from 2010 to 2100 for four selected regions (*CHN* China, *IND* India, *USA* the United States, and *E_U* European Union). Three *bars* in the box show lower quartile, median, and upper quartile. *Squares* in the box show the mean. *Lower and upper whiskers* show 10th and 90th percentile, respectively. **a** E2d, **b** E2d+10p, and **c** E45

**Fig. 10:**
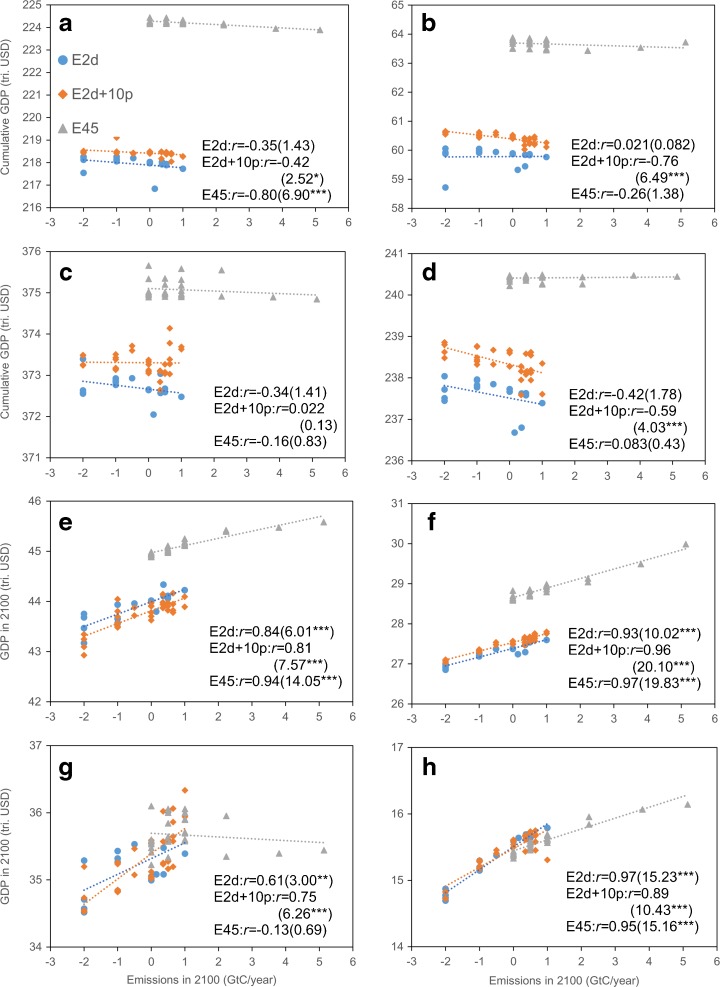
Scatter plots and correlation coefficients (*r*) between emission levels in 2100 and cumulative GDP (**a**–**d**) and GDP in 2100 (**e**–**h**). **a**, **e** China, **b**, **f** India, **c**, **g** the USA, and **d**, **h** European Union. The values in *parentheses* are *t* values for test for non-correlation (*one asterisk*, 5 % significance level; *two asterisks*, 1 % significance level; *three asterisks*, 0.1 % significance level). *Dotted lines* in the figures show approximate lines

## Appendix 1

This appendix shows the results of analyses similar to those in Section 3, particularly Section 3.2, for the four selected regions (i.e., China, India, the USA, and the European Union). These four entities are forecasted to have large economies in 2100, but the economic situation for each in the base year is different. In this appendix, we focus on the results for GDP. As Fig. 8 shows, the GDP trends shown in the four regions are similar to global-scale trends (Fig. 5). GDP in general is smaller in the three FCE scenarios (and smaller in lower cumulative emission scenarios) than in the reference scenario, although GDP continuously increases during this century. However, in the European Union, GDP declines at the end of the century in some cases. The impacts of the reduction scenarios on GDP differ by region: the largest impact is observed in India, which is around 15.5–17.8 %, while the smallest is in the USA, in which 2.5 % is the highest (both examples are in the E2d scenario).

Observing the results in more detail, first, although the cumulative emission is identical, the resulting impacts on cumulative GDP are different in the realized pathways and by region (Fig. 9). As with the impact on annual GDP, compared with the reference levels, the impact on cumulative GDP is largest in India and smallest in the USA. Furthermore, the differences by pathway are also largest in India. In the USA, there is a possibility that cumulative GDP would be greater under the E45 scenario than the reference scenario.

The two results above suggest that the Indian economy is more sensitive than that of other regions to climate mitigation and the pathways to be taken.

With regard to the emission reduction starting year (Table 4), as in the global-scale result, cumulative GDP for the two lower cumulative emission scenarios and GDP in 2100 for the lowest emission scenario are greater in all regions when emission reduction starts earlier (Table 3). However, not all the regions show statistically significant results. Significant results are observed for cumulative GDP in the USA and the European Union under the E2d and E2d+10p scenarios, while the results for GDP in 2100 are significant in India and the USA under the E2d scenario. With regard to other results, different regions show different features. For example, the results are significant for cumulative GDP under the E45 scenario in all regions except for China. However, while higher cumulative GDP is observed with earlier emission reduction starting year in the USA, the opposite results are obtained for India and the European Union. The other results were not statistically significant.

**Table 4:** Independent *t* test and one-way ANOVA applied to cumulative GDP and GDP in 2100 for different starting years of emission reduction in four selected regions

	Scenarios	Countries^a^	Average^b^	Standard deviation^b^	*t* or *F* values^c^
Cumulative GDP (5 % discount rate, trillion USD)	E2d	CHN	218.0/217.8	0.19/0.81	1.01
		IND	59.8/59.7	0.34/0.33	0.39
		USA	372.8/372.4	0.24/0.32	2.23^*^
		E_U	237.7/237.2	0.46/0.02	2.22^*^
	E2d+10p	CHN	218.4/218.4	0.21/0.090	0.23
		IND	60.4/60.4	0.15/0.17	0.80
		USA	373.4/373.1	0.26/0.0037	3.09^**^
		E_U	238.5/238.2	0.28/0.29	3.12^**^
	E45	CHN	224.2/224.2/224.3	0.057/0.092/0.15	1.56
		IND	63.5/63.7/63.8	0.031/0.069/0.046	104.06^***^
		USA	375.2/375.1/375.0	0.27/0.23/0.066	4.01^***^
		E_U	240.3/240.4/240.5	0.069/0.074/0.019	8.95^***^
GDP in 2100 (trillion USD)	E2d	CHN	43.9/43.7	0.31/0.17	1.33
		IND	27.3/27.0	0.22/0.19	2.11^*^
		USA	35.3/34.8	0.36/0.29	2.29^*^
		E_U	15.4/15.0	0.31/0.52	1.50
	E2d+10p	CHN	43.7/43.8	0.30/0.28	0.63
		IND	27.5/27.5	0.22/0.20	0.70
		USA	35.3/35.3	0.45/0.48	0.35
		E_U	15.5/15.4	0.31/0.28	1.04
	E45	CHN	45.2/45.1/45.1	0.16/0.17/0.20	1.03
		IND	28.8/28.9/28.9	0.19/0.25/0.39	0.35
		USA	35.6/35.7/35.7	0.23/0.30/0.20	0.64
		E_U	15.6/15.6/15.6	0.18/0.21/0.20	0.32

^a^ *CHN* China, *IND* India, *USA* United States, *E_U* European Union

^b^The values are in ascending order of the emission reduction starting year (the first one is the earliest). In the E45 scenario, three different starting years exist

^c^In this statistical hypothesis test, the null hypothesis is that each factor (by scenario) is the same between the two or three starting years of emission reduction. A *t* test is applied to the E2d and E2d+10 % scenarios, in which two points are compared, whereas a one-way ANOVA is applied to the E45 scenario, in which three points are compared (*one asterisk*, 5 % significance level; *two asterisks*, 1 % significance level; *three asterisks*, 0.1 % significance level)

Finally, the results for relationships with emission levels in 2100 differ by region and scenario (Fig. 10). There are moderate to strong correlations between emission levels in 2100 and cumulative GDP (Fig. 10a–d) when statistically significant results are obtained. However, about half of the results are not statistically significant. Contrary to the results for cumulative GDP, emission levels under the E45 scenario have moderate or strong positive correlations with GDP in 2100 except in the USA, where the correlation was not significant (Fig. 10e–h). These tendencies are consistent with the global-scale results (Fig. 7b).

These results suggest that the global emission pathways to realize a certain cumulate emission can differently affect regional economy (here we analyzed GDP), but not clear relationships seen in the global-scale results were observed.

## Footnotes

1 Based on USD in the base year 2001, i.e., 230 trillion USD equates to 230 trillion in 2001 constant USD
